# Circulation of multiple hepatitis B virus genotypes in individual pregnant women seeking antenatal care in northern Ghana

**DOI:** 10.1186/s12985-023-02110-2

**Published:** 2023-07-13

**Authors:** Nsoh Godwin Anabire, Osbourne Quaye, Gideon Kofi Helegbe

**Affiliations:** 1 West African Centre for Cell Biology of Infectious Pathogens (WACCBIP), Department of Biochemistry, Cell & Molecular Biology, University of Ghana, P. O. Box LG 54, Legon- Accra, Ghana; 2 Department of Biochemistry & Molecular Medicine, School of Medicine, University for Development studies, P. O. Box TL 1883, Tamale, Ghana

**Keywords:** Chronic hepatitis B, HBV genotypes, Pregnant women, Antenatal clinic

## Abstract

**Background:**

Identification and monitoring of HBV genotype variations is important, since that can help forecast the likelihood of developing serious liver disease and how well patients respond to antiviral medication. Given that HBV genotyping tests are not widely available in our healthcare system, this study characterized HBV genotypes in pregnant women seeking prenatal treatment in northern Ghana.

**Method:**

By a cross-sectional approach, 2071 pregnant women seeking antenatal care in health facilities in northern Ghana were screened for HBV infection using hepatitis B surface antigen (HBsAg) rapid diagnostic test kit. The women were aged between 17 and 41 years, were of varying gravidae (primigravidae and multigravidae) and gestational age (first, second and third trimesters). A confirmatory PCR assay was used to detect HBsAg, and the distribution of HBV genotypes was determined using a nested PCR assay.

**Results:**

Three HBV genotypes (A, D and E) were detected among the pregnant women, of which 175 (91.6%) had genotype E, 9 (4.7%) had mixed genotypes A and E, 5 (2.6%) had mixed genotypes D and E, and 2 (1.1) had mixed genotypes A, D and E. The proportions of women with the different HBV genotypes were independent of age (*p = 0.925*), gravidity (*p = 0.193, χ*^*2*^ *= 4.729*) and gestational age (*p = 0.227, χ*^*2*^ *= 8.152*).

**Conclusion:**

This study for the first-time characterized circulating HBV genotypes in pregnant women in northern Ghana, which reveals genotypes A and D are found in mixed infections with genotype E. The findings have clinical implications on the management of chronic HBV infection among pregnant women in northern Ghana.

**Supplementary Information:**

The online version contains supplementary material available at 10.1186/s12985-023-02110-2.

## Background

Nearly 296 million people throughout the world have chronic infection with hepatitis B virus (HBV) [1], which causes cirrhosis of the liver, hepatocellular cancer, and up to a million deaths per year [2]. Chronic HBV infection in Ghana is a significant public health concern that needs further attention. According to a review by Ofori-Asenso and Agyeman [3], the countrywide prevalence of HBV infection by seropositivity is 12.3%, which lists Ghana among the regions with a high prevalence (≥ 8%) of chronic HBV infection [4, 5]. A high disease burden is also seen among Ghanaian expectant mothers, with a nationwide incidence of 13.1% [3], and a prevalence of 9.2% in the northern part of the country [6].

HBV is an enveloped virus that is a member of the *Hepadnaviridae* viral family. The virus has a circular, partly double-stranded DNA molecule that is around 3.2 Kb in size [7, 8]. HBV genome exhibits great genetic variability and is divided into 9 genotypes, namely A–I; with an additional putative genotype, J, that has been identified [9–12]. HBV genotypes vary in the severity of the sickness they cause and how they respond to antiviral medication [13], and some of the genotypes are geographically restricted to particular locations while others (HBV genotypes B and D) are distributed worldwide [7]. In Southern and Northern Africa, genotypes A and D are more common, respectively [14, 15]. HBV genotype E is primarily confined and prevalent in West Africa [14], and compared to other genotypes, has not been extensively studied [16]. Although HBV infection is widespread in Ghana, little is known about the different HBV genotypes. Limited investigations have shown that the circulating genotypes in the Ghanaian population are A, D, and E, with genotype E predominating [17, 18].

A high frequency of transfer from mother to child has been linked to genotypes B, C, and I, while high transmission rates during sexual activity or drug injection has been linked to genotypes A, D and G [19, 20]. Despite this, there may not be a causal relationship between genotypes and modes of transmission given the geographic restriction of HBV genotypes. However, current meta-analysis demonstrates a general progressive decrease in the prevalence of dominant genotypes but a progressive increase in the less dominant genotypes by geographic regions, suggesting a potential association between HBV genotypes and mode of disease transmission [14]. Infections with genotypes A and C are associated with higher rates of chronification [21, 22]. Genotypes C, D, and F are assumed to be more virulent as they are associated with quicken onset of hepatocellular carcinoma and liver cirrhosis, and also exhibit higher resistance to interferon alpha and pegylated interferon alpha therapy [23–25]. Even though clinical significance of genotype E remains elusive [26, 27], the genotype has been linked to lower risks of developing cirrhosis of the liver and hepatocellular cancer [28].

It is typical for a region to have a high incidence of more than one dominant genotype, particularly for genotypes B and C in Asian-Pacific regions or genotypes A and D in Western nations [14, 29, 30]. Recombination is common, and of considerable virological and clinical relevance, when multiple distinct HBV genotypes are present in a single infection [31], which can lead to new genotypes with altered disease presentations [32, 33].

Patients with HBV infection experience significantly different clinical outcomes, which is due in part to the complicated interactions between the virus, host, and environment [34]. During chronic HBV infection and under varied conditions of selection pressures, HBV variants that can circumvent diagnostic, preventative, and therapeutic approaches can arise [13]. Considering the possibility of both short- and long-term liver disease, as well as the risk of exposing a growing fetus to teratogenic drugs, treatment of HBV infection in pregnant women should be personalized. Knowledge from this study on the circulatory HBV genotypes among pregnant women in northern Ghana will be beneficial to clinicians to appropriately manage patients, considering that HBV genotyping tests are not commonly accessible in our healthcare system.

## Methods

### Study design and population

A cross-sectional design was used for the sampling the process. Pregnant women who were seeking antenatal care services from October 2016 to February 2017 were recruited from six (6) sampling sites in northern Ghana. The sampling sites are located in Tamale metropolis of Ghana and Central Gonja District of Ghana. In the Tamale Metropolis, which is located in the Northern Region of Ghana, women were selected from Four hospitals—Bilpella Health Center, Tamale Teaching Hospital, Tamale West Hospital, and Tamale Central Hospital. In the Central Gonja District, which is located in the Savanna Region of Ghana, women were recruited from two health facilities—Sankpala Health Center and the Kosawgu Health Center. The sample size for this study is based on our previous screening data on HBV among pregnant women in the study areas [6].

### Rapid diagnostic screening of HBV infection

HBsAg test strips (Intec Products Inc., Xiamen, China) were utilized for screening of HBV infection. The sample pad of the test strip was dipped into a test tube containing 50 µL of plasma in accordance with the manufacturer’s instructions. After incubating for 15 min, results were read as either positive (two colored bands at test and control lines), negative (one colored band at control line), or invalid (no colored bands on strips), in which case the test was repeated.

### PCR confirmation of HBV infection

Using the chelex extraction procedure as previously described [6], DNA was isolated from dry blood spots of all the screened women for the confirmatory detection of HBV. HBV infection was confirmed in a PCR experiment using previously published primers that amplifies the HBV genome’s S-gene [6]. PCR products with an expected band size of 98 bp were visualized on 2% agarose gels stained with 1.5 µL ethidium bromide.

### HBV genotyping by nested PCR

Using the QIAamp DNA mini kit (Qiagen, Germany) and following the manufacturer’s instructions, DNA was extracted from sera of all participants with confirmed HBV and utilized for HBV genotyping following a nested PCR technique as previously described [35]. The first round of PCR produced a 1063 bp product of the pre-S/S region of the viral genome using universal forward and reverse primers. The PCR reaction mix contained 10 µM of each primer, 10X PCR buffer, 10 µM of deoxynucleotides and 1 U of Taq DNA polymerase (Qiagen, Germany) in a 25 µL total reaction volume. Using type-specific primers, second round of PCR was able to distinguish between six genotypes, A–F. A, B, and C genotype detection assays were multiplexed separately from genotypes D, E, and F. The PCR cycling conditions employed in the genotyping assay were the same as previously reported [35]. Using 2% agarose gels stained with 1.5 µL of ethidium bromide, PCR products were visualized, with an expected amplified product of 68 bp for genotype A, 281 bp for genotype B, 122 bp for genotype C, 119 bp for genotype D, 167 bp for genotype E, and 97 bp for genotype F.

### Statistical analysis

Data were analyzed using SPSS version 28.0 and presented as numbers and percentages. Continuous variables were compared using one-way analysis of variance (ANOVA) and presented as mean (range; minimum - maximum values). Categorical variables were compared using Pearson’s Chi-square test or Fisher’s exact test. Comparisons were considered statistically significant at 95% confidence level with a P-value < 0.05.

## Results

### Detection of HBV infection

**Fig. 1:**
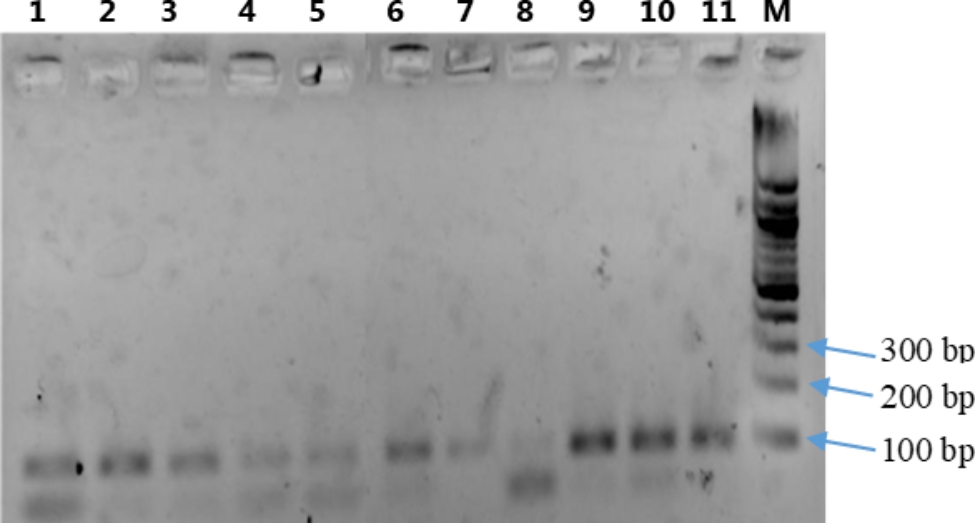
Electrophoresis of PCR products for HBV detection in pregnant women. M: 100 bp molecular weight marker (separates by 100 bp); positive samples found in wells 1–11 (98 bp). The analysis was done on 2% agarose gel

Two thousand one hundred and seventy-one (2071) pregnant women were screened for HBV infections by rapid diagnostic testing, and DNA were extracted and PCR conducted for the detection of HBV. HBV was detected in 191 (9.2%) of the pregnant women. Figure 1 shows a representative gel obtained after electrophoresis of the PCR products on a 2% agarose gel.

### Demographic information

The mean age of women diagnosed with HBV infection was 25.6 years (range: 17.0–41.0 years, n = 191). Of these women, 144 (75.4%) were multigravida while the remaining 47 (24.6%) were primigravida (Table 1). A total of 69 (36.1%) of the women were in their first trimester of pregnancy, 99 (51.8%) were in their second trimester, and 23 (12.1%) were in their third trimester (Table 1).

### Distribution of HBV genotypes

**Fig. 2:**
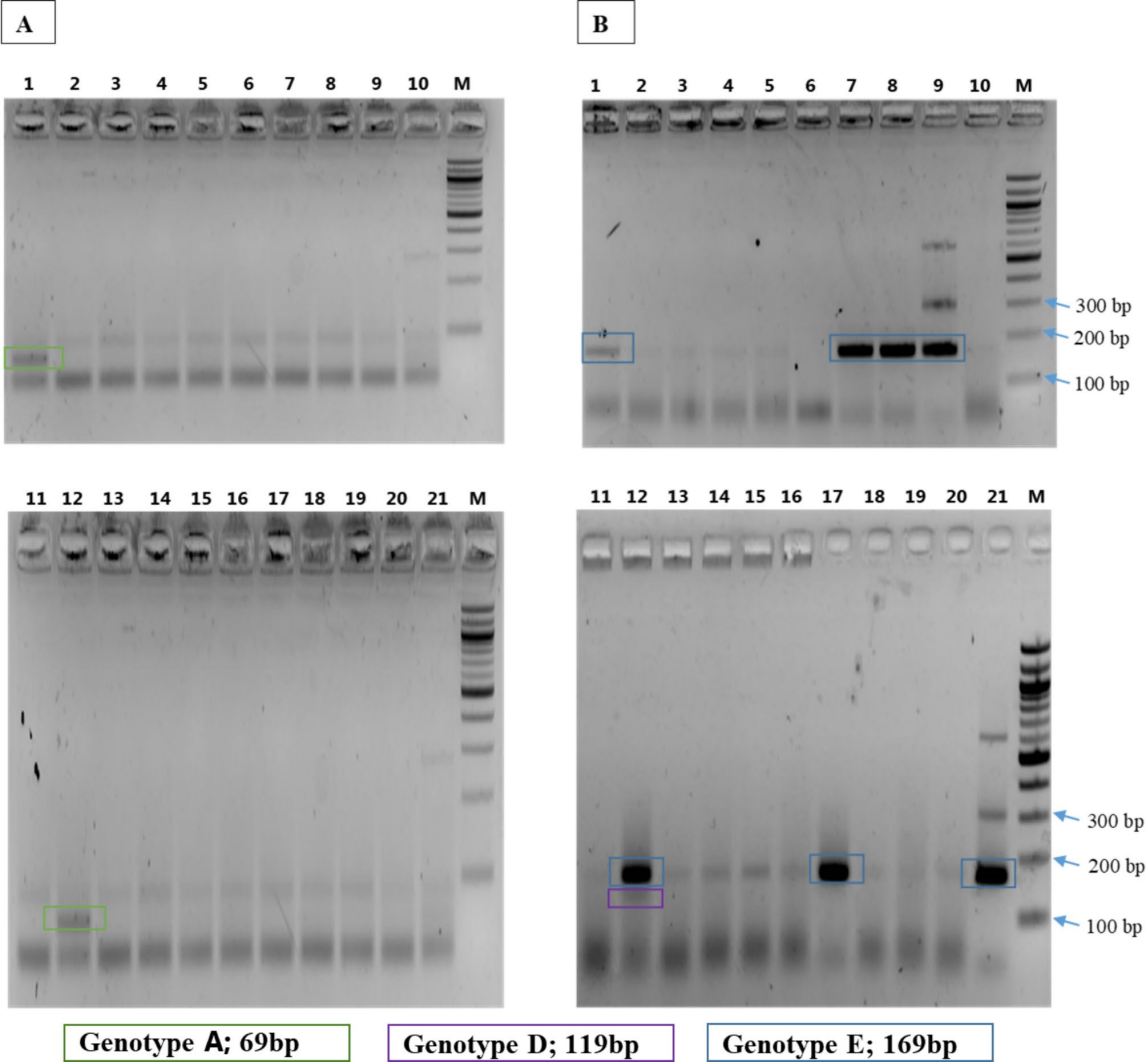
Electrophoresis of PCR products for HBV genotyping. Specific primers for **(A)** genotypes A, B and C and **(B)** genotypes D, E and F were used for the genotyping. M: 100 bp ladder (separates by a 100 bp), Wells 1–21: samples from individual pregnant women. The genotypes are identified on the gels by their respective rectangular coloured shapes as shown on the beneath of the figure. Genotypes A and D were identified as co-circulating with genotype E as represented in sample 1 (mixed genotypes A and E) and sample 12 (mixed genotypes A, D and E)

The HBV genotypes in the 191 HBV infected pregnant women were detected by nested PCR. Figure 2 shows representative 2% agarose gels after electrophoresis of the PCR products. Out of the 191 individuals, 175 (91.6%) had genotype E, 9 (4.7%) had mixed genotypes A and E, 5 (2.6%) had mixed genotypes D and E, and 2 (1.1) had mixed genotypes A, D and E. The genotypes are identified on the gels by their respective molecular weights as shown in Fig. 2. With regards to demographic and obstetric determinants, there were no age (*p = 0.925*), gravidity (*p = 0.193, χ*^*2*^ *= 4.729*) and gestational age (*p = 0.227, χ*^*2*^ *= 8.152*) differences among women with the different HBV genotypes (Table 1).

**Table 1:** Age and pregnancy history across the pregnant women with different HBV genotypes

Parameters	Single HBV genotype	Mixed HBV genotypes			*^/#^*P-value*, (χ^2^)
	E(*n* = 175)	AE(*n* = 9)	DE(*n* = 5)	ADE(*n* = 2)	
Age, years, mean (Range)	25.6 (17.0–41.0)	26.0 (21.0–33.0)	26.2 (23.0–30.0)	27.0 (21.0–33.0)	*0.925
Gravidity, n (%)					
Primigravidae	40 (22.9)	3 (33.3)	3 (60.0)	1 (50.0)	^#^0.193 (4.729)
Multigravidae	135 (77.1)	6 (66.7)	2 (40.0)	1 (50.0)	
Gestation, n (%)					
First trimester	63 (36.0)	3 (33.3)	3 (60.0)	0 (0.0)	^#^0.227 (8.152)
Second trimester	93 (53.1)	3 (33.3)	1 (20.0)	2 (100.0)	
Third trimester	19 (10.9)	3 (33.3)	1 (20.0)	0 (0.0)	

*: analyzed using one-way analysis of variance (ANOVA). #: analyzed using Chi-square or Fisher’s exact test. χ^2^: Chi-square value. *P-value* considered statistically significant at < 0.05 (2- tailed).

## Discussion

Despite the fact that HBV infection is widespread in Ghana, little is known about the different HBV genotypes. It is generally known that different HBV genotypes exhibit unique disease severity and distinct therapeutic outcomes [13]. Since HBV genotyping tests are not widely available in our health services, physicians will benefit from knowing the HBV genotypes among pregnant women as it will provide a better understanding of how to manage patients. Pregnant women in this study were found to have the HBV genotypes A, D, and E, with genotype E dominating, in line with two other investigations in Ghana [17, 18]. However, the current study found that genotypes A and D were present in mixed infections with genotype E. People with genotype D infections, in contrast to those with genotype A, are more likely to develop cirrhosis of the liver and hepatocellular carcinoma, and they are more resistant to interferon alpha and pegylated interferon alpha therapy [23–25]. Although the clinical implications of genotype E are unknown [26, 27], it has been hypothesized that genotype E may be linked to lower risks of developing cirrhosis of the liver and hepatocellular cancer [28].

It is not uncommon for a region to have a high prevalence of more than one dominant genotype, particularly for genotypes B and C in Asian-Pacific regions or genotypes A and D in Western nations [14, 29, 30]. In this study, we report of a significant number of pregnant women with coinfections of genotypes A, D and E. Our observation may be substantiated by recent meta-analysis that shows a progressive increase in the less dominant HBV genotypes by geographic regions [14]. In particular, coinfection with different HBV genotypes could lead to recombination of different viral strains [31], and such viral recombination (termed intergenotypic recombination) has been widely reported [36]. Most recombinants identified so far are of genotypes B/C or A/D hybrids, with genotypes A and C showing a higher recombination tendency compared to other genotypes [37]. New genotypes, altered clinical presentation, and disease severity can result from HBV intergenotypic recombination. An example is the most recent discovery of HBV genotype I, a unique intergenotypic recombination between genotypes A, C, and G that was discovered in Vietnam and Laos [32, 33]. Nevertheless, it is speculative to link novel HBV genotypes to different clinical presentation and the severity of liver disease. In this regard, cohort-based studies are required to substantiate the clinical relevance of new HBV genotypes when they are identified. For the evolution of HBV, recombination is crucial. Clarifying the processes of intergenotypic recombination and the clinical importance of these HBV recombinants will require more research. This current study was unable to elucidate if coinfections with genotypes A, D and E among the pregnant women could drive recombination processes and lead to viral evolution within the Ghanaian population. In this regard, sequencing of the circulatory HBV viruses will be beneficial in unraveling this effect. Such an approach will overcome the potential limitation of the genotyping approach we employed herein, which is constrained in its ability to accurately identify and categorize genotypes and variants beyond the range of the sets of genotyping primers utilized and the resolution of the agarose gel. That notwithstanding, in order to avert possible increased transmissibility of the virus due to recombination, newborn hepatitis B vaccination program should be intensified and made accessible through free health care delivery services for all newborns within the Ghanaian population. Additionally, there should be a concerted effort to educate and raise awareness on coinfection with HBV genotypes and their possible clinical implication among the Ghanaian populace.

In conclusion, we find that the predominant genotype of hepatitis B circulating in pregnant women in northern Ghana is HBV genotype E. Genotypes A and D were detected in mixed infections with genotype E. These findings have clinical implications and prompt the need to track and study the viral evolution within the Ghanaian population.

## Electronic supplementary material

Below is the link to the electronic supplementary material.

Supplementary Material 1

Supplementary Material 2

Supplementary Material 3

## Data Availability

All data supporting the findings are found in this article.

## Abbreviations

HBV: Hepatitis B virus
HbsAg: Hepatitis B surface antigen
