# Supplementary figures and images for: Circulation of multiple hepatitis B virus genotypes in individual pregnant women seeking antenatal care in northern Ghana
Source: Virol J. 2023 Jul 13;20:149. doi: 10.1186/s12985-023-02110-2 (PMC10347747; doi:10.1186/s12985-023-02110-2)

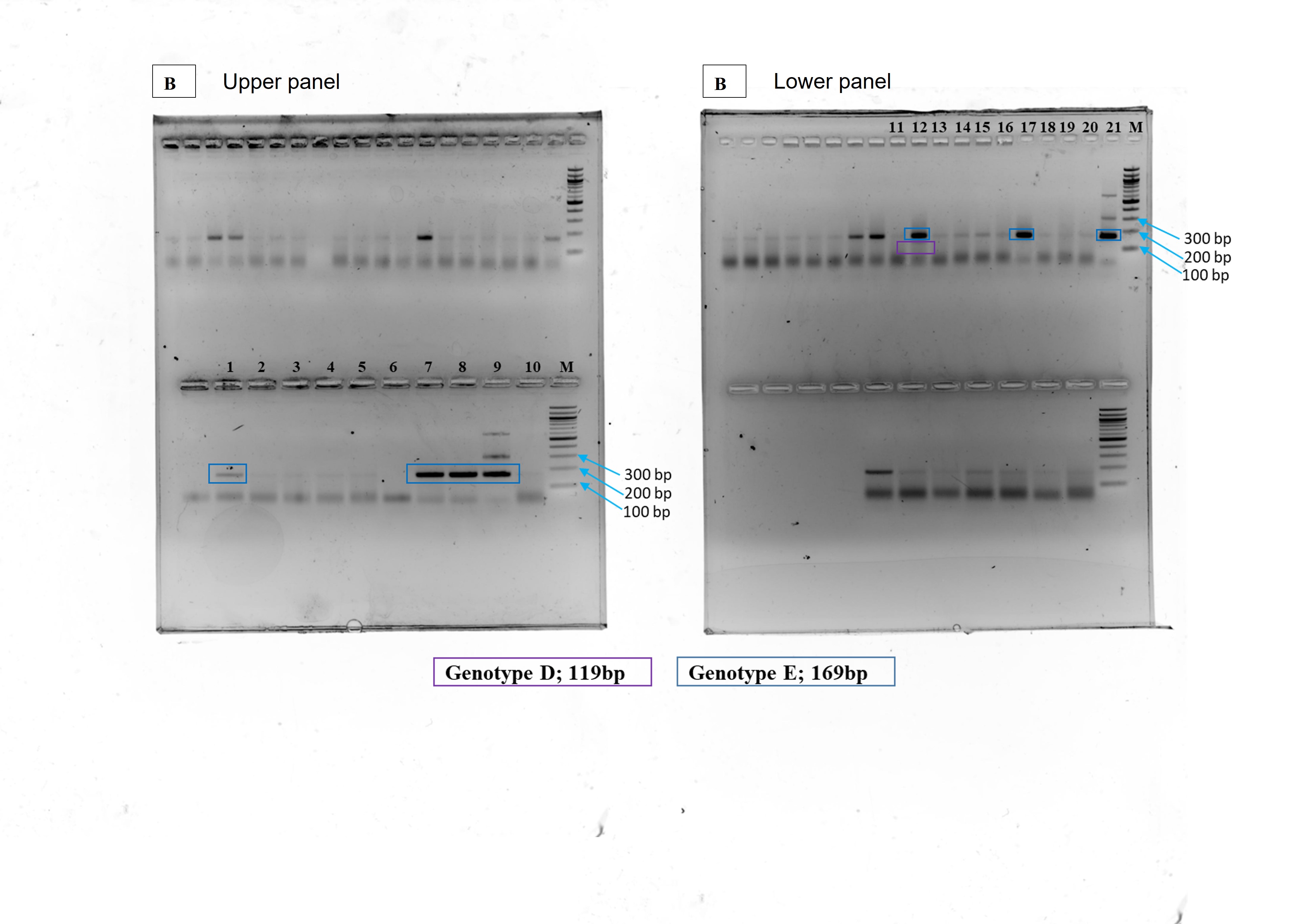

Supplement: Supplementary file 1 — Supplementary Material 1 [file 12985_2023_2110_MOESM1_ESM.jpg]

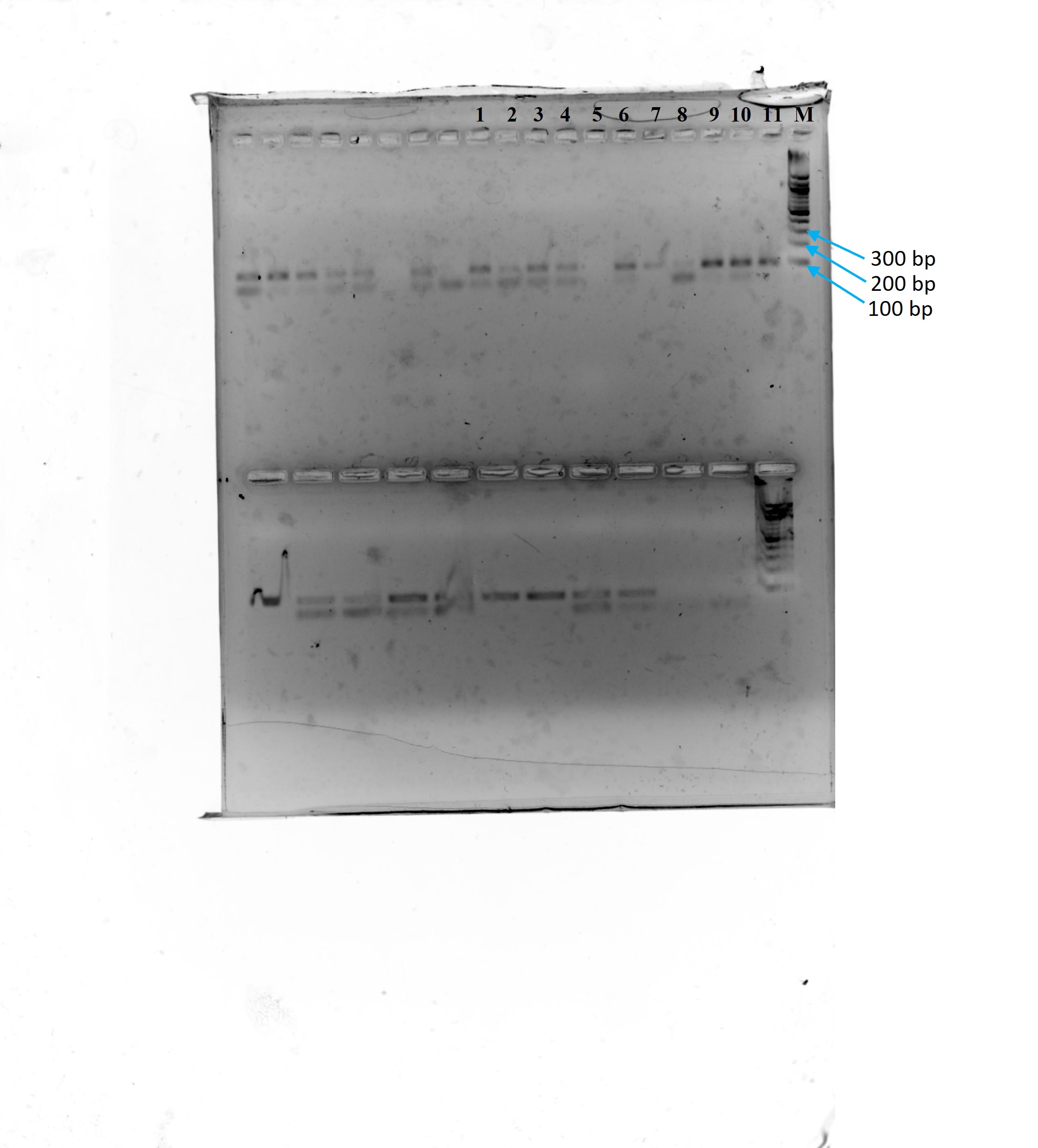

Supplement: Supplementary file 2 — Supplementary Material 2 [file 12985_2023_2110_MOESM2_ESM.jpg]

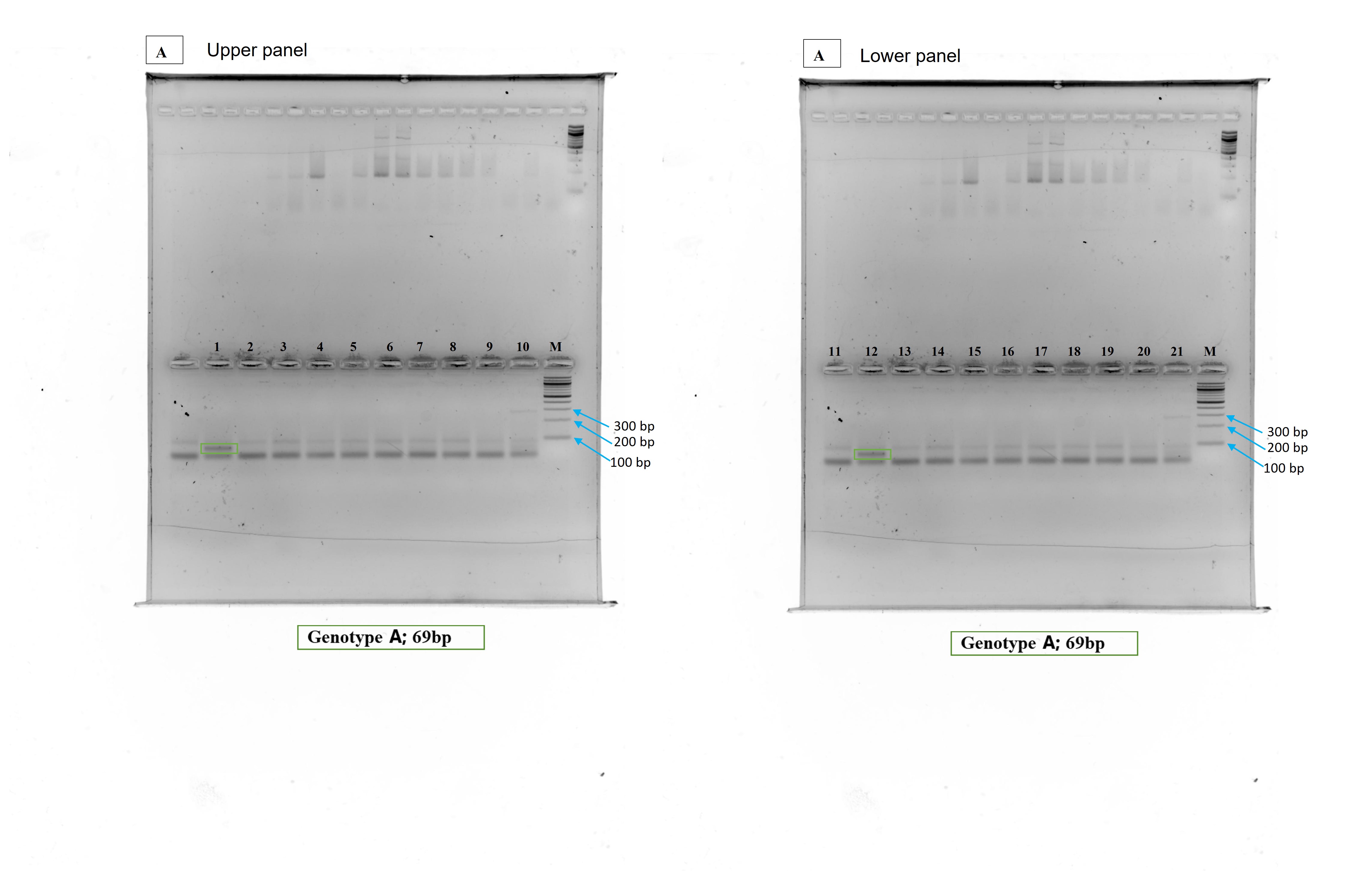

Supplement: Supplementary file 3 — Supplementary Material 3 [file 12985_2023_2110_MOESM3_ESM.jpg]
